# An investigation of the relationship between long bone measurements and stature: Implications for estimating skeletal stature in subadults

**DOI:** 10.1007/s00414-024-03336-7

**Published:** 2024-10-19

**Authors:** Elaine Y. Chu, Kyra E. Stull

**Affiliations:** 1 Department of Anthropology, Texas State University, San Marcos, TX USA; 2 Department of Anthropology, University of Nevada, Reno, Reno, NV USA; 3 Faculty of Health Sciences, Department of Anatomy, University of Pretoria, Pretoria, South Africa

**Keywords:** Stature estimation, Graphical user interface (GUI), Biological profile, Juvenile, Regression

## Abstract

The present study introduces new regression formulae that address several challenges of current subadult stature estimation methods by 1) using a large, contemporary, cross-sectional sample of subadult skeletal remains; 2) generating regression models using both lengths and breadths; 3) utilizing both linear and nonlinear regression models to accommodate the nonlinear shape of long bone growth; and 4) providing usable prediction intervals for estimating stature. Eighteen long bone measurements, stature, and age were collected from computed tomography images for a sample of individuals (*n* = 990) between birth and 20 years from the United States. The bivariate relationship between long bone measurements and stature was modeled using linear and nonlinear methods on an 80% training sample and evaluated on a 20% testing sample. Equations were generated using pooled-sex samples. Goodness of fit was evaluated using Kolmogorov–Smirnov tests and mean absolute deviation (MAD). Accuracy and precision were quantified using percent testing accuracy and Bland–Altman plots. In total, 38 stature estimation equations were created and evaluated, all achieving testing accuracies greater than 90%. Nonlinear models generated better fits compared to linear counterparts and generally produced smaller MAD (3.65 – 15.90cm). Length models generally performed better than breadth models, and a mixture of linear and nonlinear methods resulted in highest testing accuracies. Model performance was not biased by sex, age, or measurement type. A freely available, online graphical user interface is provided for immediate use of the models by practitioners in forensic anthropology and will be expanded to include bioarchaeological contexts in the future.

## Introduction

Stature is used in biological anthropology to discuss hominid evolution, evaluate human variation, assess the human condition, and aid in forensic identification. Methods for estimating stature using the skeleton have been split into two types: anatomical and mathematic [1, 2]. The anatomical method uses all bones that contribute to stature with a soft-tissue correction [3, 4]. Numerous validations of the anatomical method have achieved high accuracy [5, 6] – even in subadult groups [7]. Mathematical methods for estimating stature traditionally use one or more elements in a regression formula to estimate stature [2]. Even though they yield estimates with less precision, mathematical methods for estimating stature have persisted in biological and forensic anthropology because of ease of use, applicability on incomplete skeletons, and reliability of results [8–11].

A handful of mathematical methods for estimating stature from immature skeletal remains exist [*e.g.*, 12–18]. While this list illustrates substantial contributions to the field, these methods are similar in that they all exclusively use long bone lengths and linear regression. As such, estimating subadult stature using fragmentary remains is only possible if diaphyseal lengths are estimated [19], which could potentially compound error for the stature estimate [20]. Linear regression is widely used in adult stature estimation [*e.g.*, 21, 22], because the relationship between long bone lengths and stature is linear once skeletal maturity is reached. However, the fluctuating relationships between long bone lengths and stature complicate subadult stature estimation by necessitating nonlinear models [14, 15]. While linear regression is easy to calculate, interpret, and distribute, stature estimation methods using linear regression to appreciate a nonlinear relationship are invalid [23–26]. To the authors’ knowledge, the applicability of nonlinear regression towards improving subadult stature estimates has yet to be explored.

Current subadult stature estimation methods either have defined age ranges for their regression equations [*e.g.*, 12, 13, 15, 18] or have separate equations by chronological age [17]. Many of the defined age ranges line up with the chronological ages of different life history stages, which categorize changes in rate of growth throughout ontogeny [27, 28]. While subadult age estimation methods have increased in availability, accuracy, and precision [*e.g.*, 24, 29, 30], estimating additional aspects of the biological profile can be a source of compounding error to a stature estimate. Additionally, even the most precise age estimates extend over numerous years, limiting the utility of different methodological approaches for different ages in a real-life context. The use of nonlinear modeling can expand the applicability of subadult stature estimation to include the entire process of skeletal growth and development, which is an inherently nonlinear process [25, 26, 31].

The present study aims to address some of the limiting factors identified among current methods and improve subadult stature estimation. New regression formulae will be generated that 1) explore the utility of breadth measurements, 2) consider performance of nonlinear regression and linear regression, and 3) expand the age range. This study uses the Subadult Virtual Anthropology Database (SVAD), which is one of the largest collections of contemporary, cross-sectional subadult skeletal data currently available [32]. Large sample sizes and cross-sectional data capture a greater amount of variation resulting in more robust models [15, 25, 33]. Using the SVAD and incorporating three adjustments to regression formulae, the authors aim to introduce new equations that maintain the high accuracy of previous methods while improving upon the precision and applicability of subadult stature estimation in forensic, bioarchaeological, and paleoanthropological contexts.

## Materials and methods

A sample of 990 individuals (F = 401; M = 589) aged between birth and 20 years old from the U.S. sample of the SVAD were used for this study that is comprised of individuals from two geographically diverse locations (Baltimore, MD and Albuquerque, NM). The sample was randomly separated into a training (80%, *n* = 793) and hold-out (20%, *n* = 197) set for model creation and evaluation (Table 1). Eighteen diaphyseal measurements (see Table 2) were collected per individual, following an adapted protocol for subadult long bone measurements using CT scans [34]. Once fusion was estimated to be “active”, following the protocol outlined by Stull and Corron [34], maximum length and breadth measurements adapted from Langley and colleagues [35] were collected to include the epiphyses. The combination of diaphyseal and maximum length measurements in the sample necessitates that the measurement definitions are comparable; thus, ulna proximal breadth was excluded because there is no diaphyseal measurement for ulna proximal breadth [34]. Two additional measures were calculated by adding humerus and radius length (upper limb length) and femur and tibia length (lower limb length), following previous subadult stature estimation studies [13, 17]. Known stature, age, and sex were also retained for analyses.

**Table 1:** Summary of the 80% training and 20% testing sample used in the present study

Age (as integers)	Training (80%)		Testing (20%)		Totals
	Female	Male	Female	Male	
0	87	102	25	27	241 (24.3%)
1	29	44	7	14	94 (9.49%)
2	13	23	5	8	49 (4.95%)
3	13	16	3	5	37 (3.74%)
4	15	11	4	5	35 (3.54%)
5	14	7	3	3	27 (2.73%)
6	4	5	1	1	11 (1.11%)
7	8	8	1	2	19 (1.92%)
8	2	4	1	4	11 (1.11%)
9	6	9	0	4	19 (1.92%)
10	3	3	1	1	8 (0.08%)
11	11	6	2	3	22 (2.22%)
12	5	14	2	2	23 (2.32%)
13	8	11	3	3	25 (2.53%)
14	10	13	1	2	26 (2.63%)
15	10	31	4	7	52 (5.25%)
16	12	41	4	4	61 (6.16%)
17	15	31	4	6	56 (5.25%)
18	20	35	4	8	67 (6.77%)
19	25	35	2	4	66 (6.67%)
20	11	23	3	4	41 (4.14%)
Totals	321 (40.5%)	472 (59.5%)	80 (40.6%)	117 (59.4%)	990

**Table 2:** Long bone measurements included in the study per bone, denoted by an (x). Not all measurement types were conducted on each bone, as denoted by a (-)

Bone	Length	Proximal Breadth	Midshaft Breadth	Distal Breadth
Humerus	x	x	x	x
Radius	x	x	x	x
Ulna	x	-	x	-
Femur	x	-	x	x
Tibia	x	x	x	x
Fibula	x	-	-	-

**Fig. 1:**
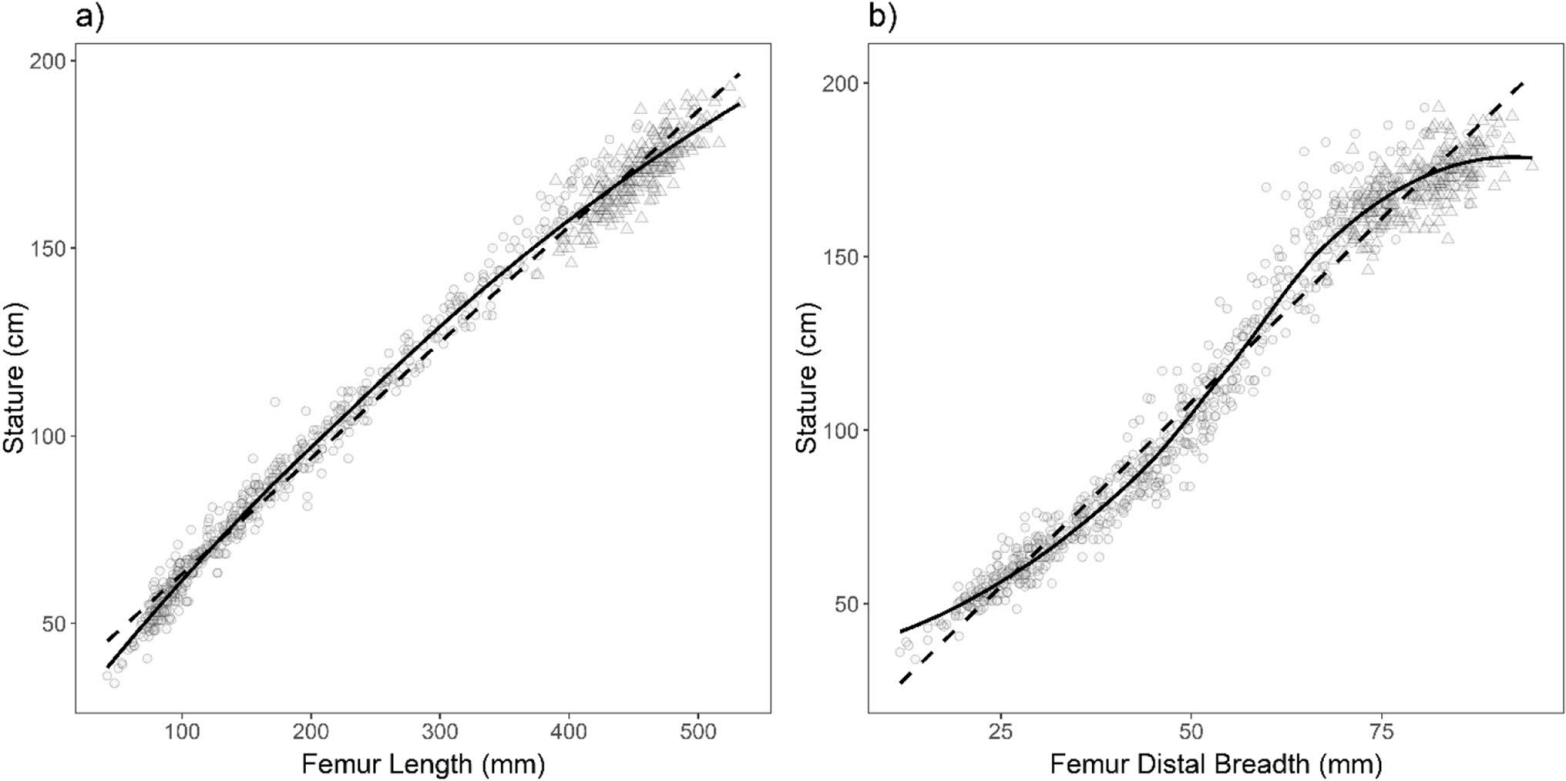
Relationship between **a**) femur length and **b**) femur distal breadth and stature. Linear relationship = dashed line. Nonlinear relationship = solid line. Diaphyseal measurements = circle. Maximum measurements = triangle

All analyses were conducted in R using RStudio [36, 37] using pooled-sex samples. The data were first tested for assumptions of normality, using a Shapiro-Wilks test due to larger sample sizes [38, 39], and linearity, using visual assessments of bivariate plots – the results of which suggested the use of nonparametric and nonlinear methods, especially for the relationship between breadth measurements and stature. Kendall’s tau correlation coefficients were calculated to explore the relationship between stature and long bone measurements as it is a nonparametric method for evaluating correlation [40, 41]. In addition to linear regression, two nonlinear regression equations commonly used in growth and development were selected (see Table 3): a three-parameter asymptotic exponential regression, which is like a power law and roughly resembles the relationship between length measurements and stature (Fig. 1a); and a three-parameter logistic regression equation, which has a sigmoidal (“S”) shape that roughly resembles the relationship between breadth measurements and stature (Fig. 1b).

**Table 3:** Linear and nonlinear regression equations used in the present study to characterize the relationship between long bone measurements and stature

Regression Type (Measurements used)	Equation
Linear (Lengths, Breadths)	y=ax+b
Three-parameter Asymptotic Exponential (Lengths)	$y=a-b{e}^{-cx}$
Three-parameter Logistic (Breadths)	$y= \frac{a}{(1+b{e}^{-cx})}$

Linear and nonlinear models were created using the *stats* package [36]. After model creation, resulting in a regression and 95% prediction intervals using the training set, each model was validated using the 20% testing sample to assess the generalizability of the models on separate data. Goodness of fit of linear and nonlinear models were evaluated using residual standard deviation [42], Kolmogorov–Smirnov tests of equal distributions [43] comparing known stature and predicted stature, and mean absolute deviation (MAD) exploring the level of precision provided by each regression. These measures were chosen to evaluate model performance because traditional measures of goodness-of-fit for linear regression, such as an adjusted R^2^, cannot be directly applied to nonlinear models [44]. In addition, test accuracy was calculated to evaluate the overall accuracy and generalizability of the model by demonstrating whether the model can still provide a 95% PI that encapsulates the known stature of an individual even when the individual is not included in the training sample. Bland–Altman plots were also used to evaluate the magnitude and direction by which a model over- or under-estimates stature compared to known stature [45].

## Results

Kendall’s tau correlations inspecting the relationship between all long bone measurements and stature ranged from 0.759 – 0.907 (Tables 4 and 5). In general, lengths were more strongly correlated with stature than breadths, while proximal and distal breadths were more strongly correlated to stature than midshaft breadths. Combining proximal and distal limb lengths did present with higher correlations to stature (upper limb length: 0.907; lower limb length 0.904). In total, 38 out of the 40 expected models were created for stature estimation; nonlinear models for tibia distal breadth and radius distal breadth failed to reach convergence. The coefficients of all models created were statistically significant (*p* < 0.001). Performance metrics for all linear and nonlinear pooled models are summarized in Tables 4 and 5 and are organized by long bone and measurement.

**Table 4:** Summary of linear and nonlinear regression equations and Kendall’s tau correlation coefficients (*r*) for the relationship between each long bone measurement and stature in the upper limb. The *p*-values for all coefficients were statistically significant, with *p* < 0.001

Long Bone	Measurement Type	Kendall’s Tau Correlation (*r*)	Model Type	Equation	Residual Standard Deviation (in cm)	Kolmogorov–Smirnov Test Statistic (*D*)	Test Accuracy (in %)	Mean Absolute Deviation (MAD)
Humerus	Length	0.905	Linear	y=0.46x+25.3	5.56	0.0833	95.8	5.54
			Nonlinear	$y=337-329{e}^{-0.00214x}$	4.60	0.0476	95.8	3.99
	Proximal Breadth	0.830	Linear	y=3.97x-3.93	8.84	0.0544	96.6	8.31
			Nonlinear	$y= \frac{231}{\left(1+9.81{e}^{-0.0758x}\right)}$	8.27	0.0544	95.9	7.75
	Midshaft Breadth	0.820	Linear	y=8.22x-6.71	13.5	0.114	97.2	10.2
			Nonlinear	$y= \frac{211}{\left(1+12.2{e}^{-0.184x}\right)}$	12.1	0.0909	94.3	9.98
	Distal Breadth	0.854	Linear	y=2.88x-2.21	9.02	0.0838	93.9	8.22
			Nonlinear	$y= \frac{198}{\left(1+10.6{e}^{-0.0716x}\right)}$	7.30	0.0503	95.0	7.03
Radius	Length	0.895	Linear	y=0.610x+24.4	6.23	0.0888	98.8	6.14
			Nonlinear	$y=297-295{e}^{-0.00352x}$	4.77	0.0533	97.0	4.39
	Proximal Breadth	0.826	Linear	y=8.16x+1.01	10.5	0.0686	94.9	7.57
			Nonlinear	$y= \frac{196}{\left(1+11.6{e}^{-0.021x}\right)}$	8.38	0.0629	91.4	7.84
	Midshaft Breadth	0.808	Linear	y=12.1x-13.3	13.6	0.0994	94.2	12.3
			Nonlinear	$y= \frac{208}{\left(1+13.9{e}^{-0.273x}\right)}$	12.5	0.0819	93.0	11.9
	Distal Breadth	0.810	Linear	y=5.41x+1.37	11.7	0.100	93.3	9.04
			Nonlinear	-	-	-	-	-
Ulna	Length	0.895	Linear	y=0.581x+21	5.64	0.0702	97.7	5.71
			Nonlinear	$y=338-336{e}^{-0.00268x}$	4.62	0.0585	96.5	3.88
	Midshaft Breadth	0.759	Linear	y=13.1x-18.3	17.3	0.124	94.7	15.4
			Nonlinear	$y= \frac{209}{\left(1+15.7{e}^{-0.295x}\right)}$	16.3	0.101	95.3	15.9
Upper Limb (Humerus + Radius)	Length	0.904	Linear	y=0.262x+24.8	5.52	0.0864	99.4	5.52
			Nonlinear	$y=325-319{e}^{-0.00129x}$	4.44	0.0494	96.9	3.93

**Table 5:** Summary of linear and nonlinear regression equations and Kendall’s tau correlation coefficients (*r*) for the relationship between each long bone measurement and stature in the lower limb. The *p*-values for all coefficients were statistically significant, with *p* < 0.001

Long Bone	Measurement Type	Kendall’s Tau Correlation (*r*)	Model Type	Equation	Residual Standard Error (in cm)	Kolmogorov–Smirnov Test Statistic (*D*)	Test Accuracy (in %)	Mean Absolute Deviation (MAD)
Femur	Length	0.905	Linear	y=0.309+32.2	5.29	0.0778	96.1	5.10
			Nonlinear	$y=366-345{e}^{-0.00127x}$	4.49	0.050	95.6	4.06
	Midshaft Breadth	0.839	Linear	y=6.84x-3.28	9.88	0.0889	95.6	7.31
			Nonlinear	$y= \frac{209}{\left(1+10.8{e}^{-0.153x}\right)}$	8.71	0.0611	96.7	3.63
	Distal Breadth	0.871	Linear	y=2.12x+2.04	8.95	0.0942	94.8	7.61
			Nonlinear	$y= \frac{217}{\left(1+9.35{e}^{-0.0447x}\right)}$	7.87	0.0733	95.8	6.96
Tibia	Length	0.905	Linear	y=0.372x+32.1	5.65	0.0829	97.8	5.65
			Nonlinear	$y=328-311{e}^{-0.00181x}$	4.59	0.0522	96.1	3.82
	Proximal Breadth	0.869	Linear	y=2.17x+17.0	9.74	0.0733	92.7	7.51
			Nonlinear	$y= \frac{198}{\left(1+7.47{e}^{-0.0545x}\right)}$	7.90	0.0733	93.7	7.75
	Midshaft Breadth	0.819	Linear	y=7.42x+0.409	10.9	0.0659	95.6	7.92
			Nonlinear	$y= \frac{206}{\left(1+10.1{e}^{-0.017x}\right)}$	9.61	0.0549	93.4	8.09
	Distal Breadth	0.858	Linear	y=3.07x+18.4	10.8	0.0785	95.8	8.34
			Nonlinear	-	-	-	-	-
Fibula	Length	0.907	Linear	y=0.377x+33.0	5.71	0.082	97.3	4.85
			Nonlinear	$y=339-320{e}^{-0.00173x}$	4.76	0.0601	96.7	3.65
Lower Limb (Femur + Tibia)	Length	0.907	Linear	y=0.169x+32.1	5.19	0.0791	96.6	5.22
			Nonlinear	$y=351-332{e}^{-0.000734x}$	4.28	0.0508	94.9	3.76

### Goodness of fit

The goodness of fit metrics both demonstrate generally better fits using nonlinear regression compared to linear regression (Tables 4 and 5). Residual standard deviation values are consistently lower for nonlinear models compared to their linear counterparts, demonstrating a better fit of the nonlinear regression equations for both breadth and length measurements. Similar patterns are found when evaluating MAD, except for ulna midshaft breadth, which yielded the poorest fits for both linear (MAD = 15.40cm; *D* = 0.124) and nonlinear models (MAD = 15.90cm; *D* = 0.101). MAD results yielded smaller values for nonlinear regression (3.65 – 11.90cm) compared to linear regression models (4.85 – 12.30cm). Within nonlinear models, length models resulted in the lowest MAD values (3.65 – 4.39cm), followed by distal (6.96 – 7.03cm), proximal (7.75 – 7.84cm), and finally midshaft breadth (8.09 – 15.90cm). For linear models, while length models also resulted in the lowest MAD values (4.85 – 6.14cm), the subsequent order of precision of breadth models is less patterned.

All Kolmogorov–Smirnov tests yielded *p*-values above the threshold of 0.05, suggesting an overall good fit attained by the regression equations, regardless of model or measurement type. However, when evaluating model fits based on increasing values of the actual test statistic, *D*, which is a generalization of the distance between point estimates and known stature, nonlinear models tend to outperform their linear counterparts. There is also no discernible pattern in the goodness of fit, per the Kolmogorov–Smirnov tests, between length models versus breadth models (Tables 4 and 5).

### Model performance

**Fig. 2:**
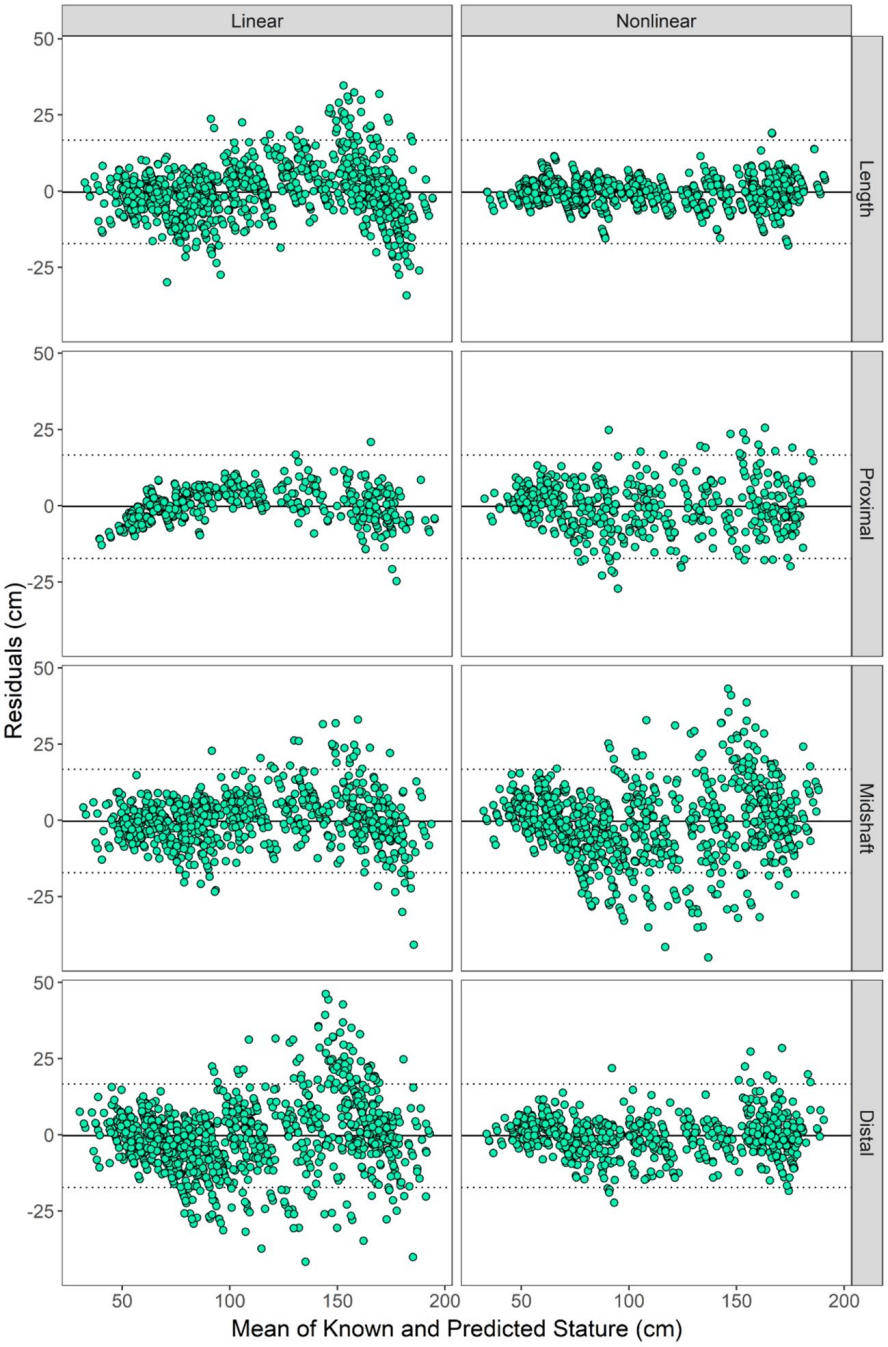
Bland–Altman plots demonstrating the pattern of residuals based on measurement type (rows) and model type (columns). The solid line represents the mean residuals, while the dashed lines represent the upper and lower bounds of a 95% confidence interval

Testing accuracy for the nonlinear models ranged from 91.4 – 97.0%, while testing accuracy for linear models ranged from 92.7 – 99.4% (Tables 4 and 5). 25 out of the 40 models created (62.5%) achieved at or above 95% testing accuracy. In general, length models yielded higher testing accuracy than breadth models, although some breadth models yielded high testing accuracy (*e.g.*, nonlinear femur midshaft breadth at 96.7%) but also reported low precision (MAD = 8.10cm). Bland–Altman plots (Fig. 2) demonstrate greater precision of the nonlinear models for length and distal breadth measurements and greater precision for linear models for proximal and midshaft breadth measurements. However, the linear models display patterned biases compared to their nonlinear counterparts. Residuals for the linear proximal breadth models demonstrates a pattern of over-estimation of stature for smaller (30 – 70cm) individuals and under-estimation of stature for midsize (70 – 150cm).

### Model misclassifications

**Fig. 3:**
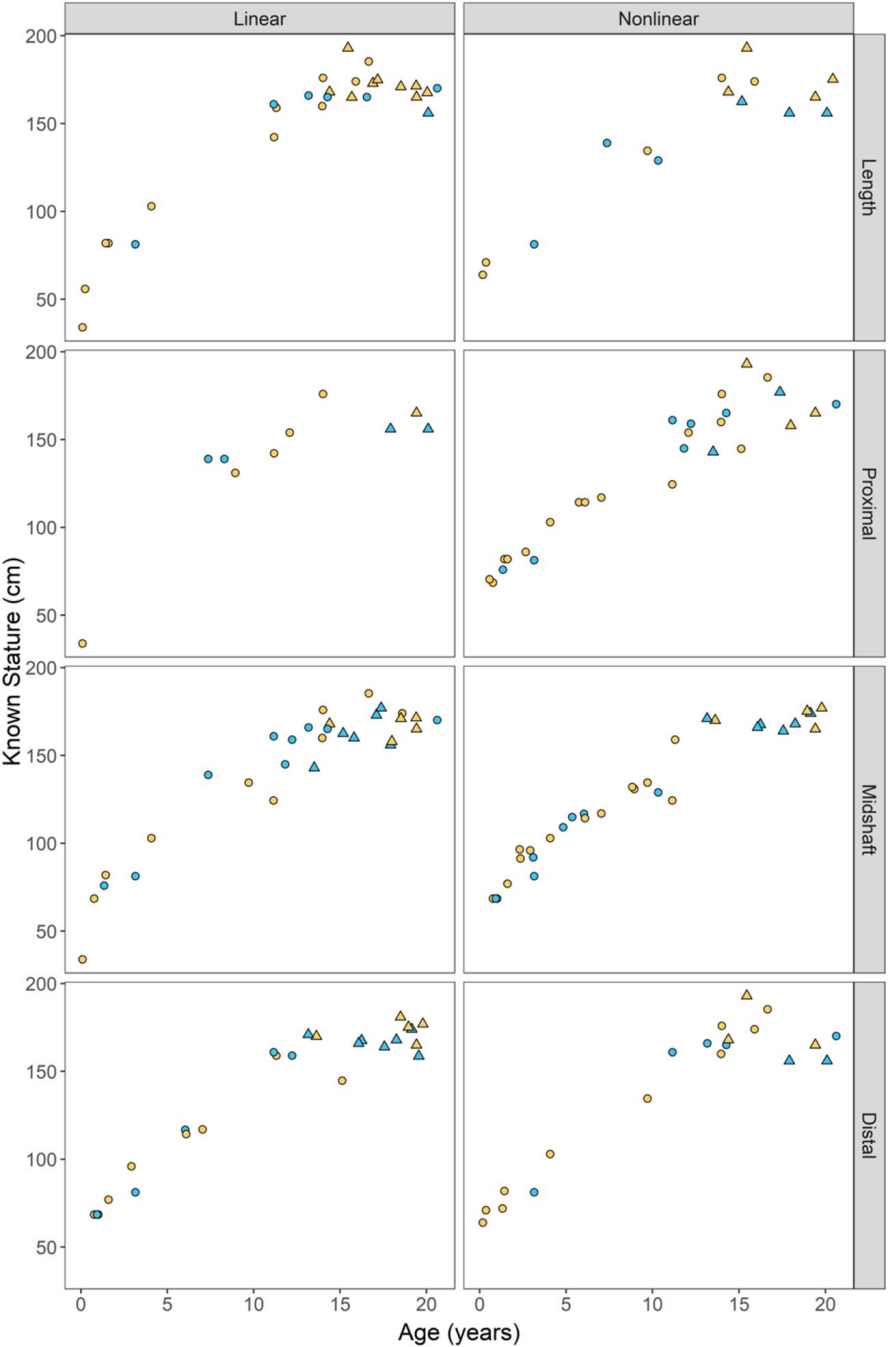
Misclassifications by measurement type (rows) and model type (columns). Female = filled blue shapes. Male = filled yellow shapes. Diaphyseal measurements = circles. Maximum measurements = triangles

From the testing set (*n* = 197), 78 individuals had at least one inaccurate stature prediction, as defined as known stature not falling within the 95% PI, generated by one of the 38 linear and nonlinear models. A summary of misclassifications by model and measurement type is provided in Table 6. Out of 6675 total stature predictions generated for this study, 296 (4.43%) resulted in misclassifications. Of those misclassifications, linear models accounted for 49.0% of the misclassifications, while nonlinear models accounted for 51.0% of the misclassifications. It is worth noting that while methods using breadth measurements may appear to yield lower misclassifications, and therefore higher accuracy, this is because there are overall less breadth measurements in the testing sample to use for predictions. The MAD values and testing accuracy for length models, both linear and nonlinear, still outperformed those of breadth models (Tables 4 and 5). Investigations into the demographics of the misclassified individuals yielded no biases by sex, age, or measurement type (Fig. 3); instead, these individuals tended to have atypical long bone measurements compared to stature.

**Table 6:** Percent of misclassifications by measurement type and model type among all predictions. Percent among all misclassifications are provided in parentheses

	Length	Proximal	Midshaft	Distal	Totals
Linear	0.78 (17.6)	0.19 (4.39)	0.58 (13.2)	0.61 (13.9)	2.17 (49.0)
Nonlinear	0.58 (13.2)	0.49 (11.1)	0.72 (16.2)	0.46 (10.5)	2.26 (51.0)
Totals	1.36 (30.8)	0.68 (15.5)	1.3 (29.4)	1.07 (24.4)

## Discussion

The present study is novel in that it 1) introduces equations that predict stature using breadth measurements, 2) is the first to use nonlinear regression to characterize the relationship between long bone measurements and stature, 3) provides subadult stature estimation equations that can be used for a wider range of ages, and 4) provides usable prediction intervals for researchers and practitioners. Over half (25 out of 38) of the stature estimation equations presented in this study achieved testing accuracy of greater than 95%. Of those models, nine models – one linear and eight nonlinear – report MAD values under 5cm, demonstrating high precision. Accuracy and precision are important factors for choosing an appropriate model for estimating aspects of the biological profile in forensic anthropology [46–49]. However, the same standards are equally considered in estimating stature in bioarchaeology, where stature is used to evaluate the human condition [50–52], and paleoanthropology, where interest in stature is mainly rooted in questions of overall body size [53, 54].

### Why breadths?

Results in this study demonstrate the utility of breadth measurements to estimate subadult stature when length measurements are not available. Of the 22 breadth regression equations generated in this study, 10 achieved a testing accuracy greater than 95%. Breadth models (MAD range: 6.96 – 15.9cm) are consistently less precise than length models (MAD range: 3.65 – 6.14cm). The smallest measurements (midshaft breadths) produced stature estimates that were less precise than their proximal and distal breadth counterparts, a phenomenon previously described for subadult age estimation using long bone measurements [25]. The reduced precision achieved by the breadth regression equations is consistent with the varied relationship (and lower values of *r*) between breadth measurements and stature as compared to length measurements and stature. Results of the Kolmogorov–Smirnov test report 14 of the 22 breadth models yield D values comparable to length models, suggesting that both linear and nonlinear regression were able to model the relationship between breadth measurements and stature with similar fits to models using length measurements. From the Bland–Altman plots (Fig. 2), several breadth measurement models (both linear and nonlinear) yielded more precise and less biased stature predictions compared to predictions generated by the linear length model.

While breadth regression equations cannot compete with length regression equations, it is important to contextualize the utility of breadth measurements in forensic, bioarchaeological, and paleoanthropological contexts. The strongly correlated relationship between long bone lengths and stature has long been taken for granted in biological anthropology, as evidenced by the exclusive availability of subadult stature estimation methods only using long bone lengths [*e.g.*, 12, 13, 17, 18. Models using breadth measurements were included in the current study to maximize the applicability of regression equations to include contexts where preservation may be poor [55, 56]. While a few methods for estimating adult stature using breadth measurements exist [56–58], to the authors’ knowledge, stature estimation methods using breadth measurements have yet to be created for immature remains until the current study.

### On nonlinearity

The relationship between age, long bone measurements, and stature is inherently intertwined in subadult research. The choice to include breadth measurements as methods for estimating subadult stature necessitated the use of nonlinear regression. A three-parameter logistic regression equation was chosen to characterize the sigmoidal shape that is represented by the relationship between breadth measurements and stature for subadults (Fig. 1b). However, what about long bone lengths? The nonlinear relationship between age and long bone lengths [25, 59] and stature [60–62] in growth and development are well established. The third member in this strongly correlated trifecta is the relationship between long bone lengths and stature, which allows for the construction of stature estimation methods [*e.g.*, 12–18. The question we ask in this study is: if the other two sides of the relationship are nonlinear, why has the relationship between long bone length and stature been consistently represented as linear during growth and development?

The major tenant of linear regression is the assumption of linearity – and while we, and previous methods of stature estimation, have demonstrated that the relationship between long bone lengths and stature is quite linear (Fig. 1a), the tail ends of growth and development necessitate a consideration of a not-quite-linear relationship. Results of the present study demonstrate that nonlinear models, using the three-parameter asymptotic exponential regression equation, result in greater goodness of fit of the relationship between long bone length and stature, as evidenced by the results of the Kolmogorov–Smirnov tests and MAD (Tables 4 and 5). In both cases, smaller values of *D* or MAD represent a closer relationship between the known stature and point estimates produced by the nonlinear models compared to their linear counterparts. The greater goodness of fit of the nonlinear models using length measurements is best appreciated when known stature is between 50 – 100cm (Fig. 2), which covers the first three years after birth. In general, the linear length models in the present study over-estimate stature for individuals within this age range, especially compared to the nonlinear length models (Fig. 2). This period of growth, otherwise known as the infant life history stage, is characterized by rapid growth prior to deceleration in growth velocity during childhood and through adolescence [27, 28]. Murray and colleagues [15] recently also demonstrated that the ratio between femur or humerus length and stature exhibits nonlinear changes that are especially apparent during the first three years of life in their sample, further supporting the application of nonlinear regression to capturing the relationship between long bone lengths and stature.

### Coverage in subadult methods

**Fig. 4:**
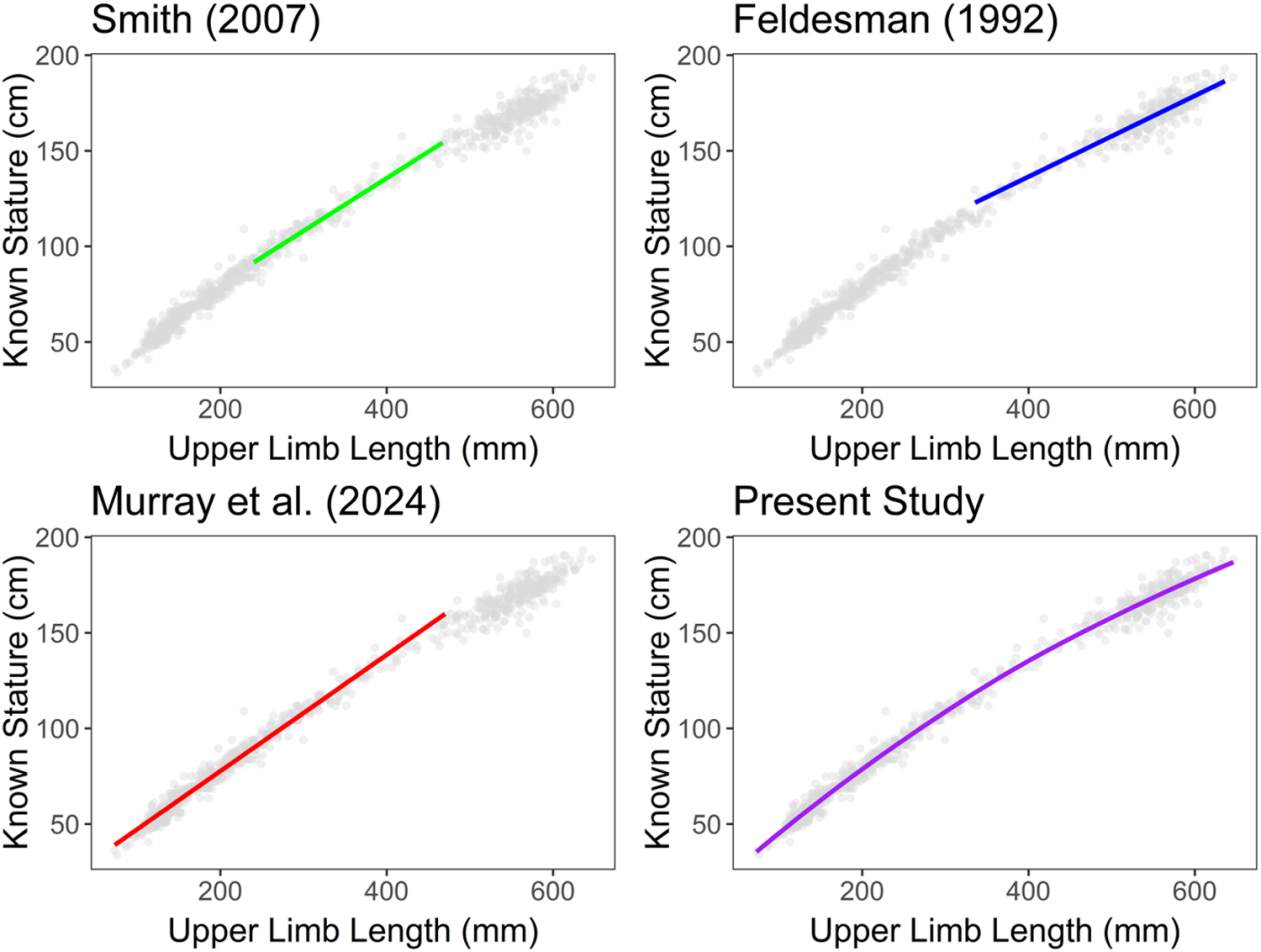
Coverage of different stature estimation methods compared to the current study

Other methods of subadult stature estimation have addressed the nonlinearity of the relationship between long bone lengths and stature by generating multiple linear equations by age cohorts [17] or truncating age ranges [14, 15, 18]. Not only does truncating age ranges reduce the nonlinearity of the relationship between long bone lengths and stature, but it also requires an a priori knowledge of age and excludes methods for certain individuals who may be classified as “subadult” (see examples in Fig. 4). While it is widely accepted that subadult age estimation methods remain the most precise in biological anthropology [29, 59, 63, 64]. Yet, the age ranges generated by these methods begs the following questions: Should a stature estimation method be used when the individual might be outside of the age range of reference material? Do you use multiple age-specific methods to estimate stature? How do you combine the resulting point estimates and prediction intervals? The present study addresses these questions by using reference material that spans almost the entirety of growth and development, from birth to 21 years of age.

### KidStats: Stature

**Fig. 5:**
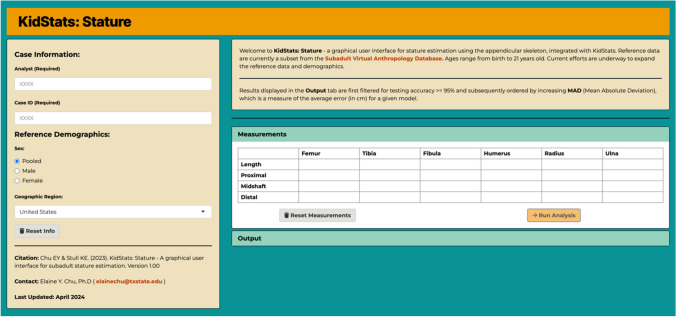
Landing page of “KidStats: Stature”

Stature estimates in a forensic biological profile should be provided as a range provided by a 90% PI, or greater [33, 65]. None of the currently available methods for estimating stature from immature remains provide guidance for calculating the 90 – 95% PI for the equations from the data given. Guidance on how to calculate confidence intervals are provided by some methods [*e.g.*, 15; however, confidence intervals are only applicable to the regression itself, including the data used to create a regression, whereas prediction intervals are what should be used when introducing new data (*i.e.*, future predictions) [66]. PIs can be calculated for linear models by hand if sample size, mean and standard deviation of the input variable, and model standard error of estimate (SEE) are provided. However, the calculation for a PI from a nonlinear equation is even more complex and the tedium of calculations using complex nonlinear equations prevents ease of use for biological anthropologists. To address these barriers to subadult stature estimation, a graphical user interface (GUI) called “KidStats: Stature” was created (Fig. 5). A direct link to the stature estimation GUI is available at the following URL: https://elaineychu.shinyapps.io/ks-stature. KidStats: Stature contains all 38 linear and nonlinear stature estimation models from the current study, allows a practitioner to enter in all available long bone measurements, and returns stature estimates using all provided estimates. The GUI also provides the user with a PDF record of the stature estimation analysis to be used for recordkeeping. While the authors only recommend the use of KidStats: Stature for forensic anthropology casework in the U.S., as the only available reference sample – as of yet – consists of cross-sectional, contemporary data from the U.S., additional uses will become available as more reference data are added.

### Limitations and future considerations

While the present study has attempted to address the limitations of previous studies regarding the relationship between long bone measurements and stature, additional considerations must be made for future studies. First, while the data used in this study were separated into 80% training and 20% testing samples prior to model construction, the testing sample does technically still belong to the same reference source even if it is comprised of geographically diverse individuals [32]. Future validation of the linear and nonlinear regression equations in this study may then benefit from testing using data from a completely different source, such as a different country or temporal period, to better understand the generalizability of these models beyond the context of forensic casework in the United States. Second, stature information for this dataset is comprised of a mixture of cadaveric and living stature obtained from next-of-kin interviews. It has been previously found that differences between living and cadaveric stature exist and can affect stature predictions [67, 68]. However, current recommendations for addressing the discrepancies between cadaveric and living stature are to simply report prediction intervals in lieu of point estimates [5, 65, 67]. Third, the choice to create models based on pooled-sex samples was somewhat deliberate, to remove the requirement of sex estimation prior to stature estimation. While there are documented differences in sexual size dimorphism in humans, the degree of dimorphism varies globally [69–71] and the effects of sexual size dimorphism on subadult stature estimates should be evaluated in greater detail. Previous literature investigating the role of sex in subadult stature estimation suggest sex-specific methods are preferrable [13, 18] and future research using the same methods as the current study will aim to address this concern by creating sex-specific models.

An additional point of consideration for the present study is the sample demographics themselves – while the sample size in the current study considerably larger than that of previous methods using cross-sectional data for subadult stature estimation [*e.g.*, 12, 13, 15] there are differences in sample size across the age distribution (Table 1). There is an underrepresentation of individuals aged between six and fourteen and an overrepresentation of individuals at birth (age at zero). This imbalance of sample sizes by age may result in decreased accuracy of stature estimates for individuals aged between six and fourteen, although results of the present study does not indicate any biases in stature estimation based on age (Fig. 3). The authors of this study also hope to apply the same research workflow to bioarchaeological reference material, to gain greater insight into the effects of secular change on body proportions and to provide stature estimation equations that are accurate, precise, and demographically applicable for bioarchaeology.

## Conclusion

The present study addresses some of the past limitations to subadult stature estimation research by testing the utility of nonlinear regression and breadth measurements to estimate stature in growing individuals (i.e., subadults) and demonstrating the utility of linear and nonlinear regression equations beyond prediction and into interpretation. This study demonstrates how treating the relationship between long bone lengths and stature as nonlinear results in models that fit the data better, resulting in higher precision while also maintaining high accuracy. The consideration of breadth measurements as alternatives for stature estimation in situations of poor preservation and/or fragmentary remains expands the applicability of the methodology beyond forensic contexts and into bioarchaeology and paleoanthropology. The use of a large, diverse reference sample additionally provides forensic anthropologists with updated regression equations for estimating stature, whether using linear or nonlinear models, that better reflect the demographics of forensic casework in the United States, while providing a framework for similar studies to be conducted using skeletal reference data that are demographically and temporally diverse. The absence of age and sex biases in the nonlinear regression equations also suggests that these methods may be used without prior estimation of age or sex, thus reducing the potential for compounding error. Finally, integration of the new regression formulae into a freely available GUI expands the applicability of nonlinear models to subadult stature estimation. Future research will focus on expanding reference samples for global and population-specific methods and generate subadult stature estimation methods for historic remains.

## Data Availability

The datasets and all analyses used/generated through this project are freely available on GitHub at: https://github.com/ElaineYChu/chu-and-stull_implications-subadult-stature. The analyses are further provided and explained at: https://rpubs.com/elainechu/chu-and-stull_implications-subadult-stature.

## References

[1] Thomas D (1894) Methods of estimating the height from parts of the skeleton: MALE FEMALE. Med Rec (1966-1922) 46:293. Retrieved from https://www.proquest.com/scholarly-journals/methods-estimting-height-partsskeleton/docview/230878929/se-2

[2] Lundy JK (1985) The mathematical versus anatomical methods of stature estimate from long bones. Am J Forensic Med Pathol 6:73–76. 10.1097/00000433-198503000-00013 3984986 10.1097/00000433-198503000-00013

[3] Fully G (1956) Une nouvelle méthode de détermination de la taille. Ann Med Legale 35:266–273 13395069

[4] Raxter MH, Auerbach BM, Ruff CB (2006) Revision of the Fully technique for estimating statures. Am J Phys Anthropol 130:374–384. 10.1002/ajpa.20361 16425177 10.1002/ajpa.20361

[5] Langley NR (2017) Stature estimation. In: Langley NR, Tersigni-Tarrant MTA (eds) Forensic anthropology: A comprehensive introduction, 2nd edn. CRC Press, pp 195–204

[6] Niskanen M, Maijanen H, McCarthy D, Junno J-A (2013) Application of the anatomical method to estimate the maximum adult stature and the age-at-death stature. Am J Phys Anthropol 152:96–106. 10.1002/ajpa.22332 23907777 10.1002/ajpa.22332

[7] Brits DM, Manger PR, Bidmos MA (2018) Assessing the use of the anatomical method for the estimation of sub-adult stature in Black South Africans. Forensic Sci Int 283:221.e1-221.e9. 10.1016/j.forsciint.2017.11.024 29258721 10.1016/j.forsciint.2017.11.024

[8] Duyar I, Pelin C (2003) Body height estimation based on tibia length in different stature groups. Am J Phys Anthropol 122:23–27. 10.1002/ajpa.10257 12923901 10.1002/ajpa.10257

[9] Formicola V (1993) Stature reconstruction from long bones in ancient population samples: An approach to the problem of its reliability. Am J Phys Anthropol 90:351–358. 10.1002/ajpa.1330900309 8460658 10.1002/ajpa.1330900309

[10] Maijanen H (2009) Testing anatomical methods for stature estimation on individuals from the W. M. Bass Donated Skeletal Collection. J Forensic Sci 54:746–752. 10.1111/j.1556-4029.2009.01053.x 19486438 10.1111/j.1556-4029.2009.01053.x

[11] Mays S (2016) Estimation of stature in archaeological human skeletal remains from Britain. Am J Phys Anthropol 161:646–655. 10.1002/ajpa.23068 27535104 10.1002/ajpa.23068

[12] Abrahamyan DO, Gazarian A, Braillon PM (2008) Estimation of stature and length of limb segments in children and adolescents from whole-body dual-energy X-ray absorptiometry scans. Pediatr Radiol 38:311–315. 10.1007/s00247-007-0720-x 18196233 10.1007/s00247-007-0720-x

[13] Brits DM, Bidmos MA, Manger PR (2017) Stature estimation from the femur and tibia in Black South African sub-adults. Forensic Sci Int 270:277.e1-277.e10. 10.1016/j.forsciint.2016.10.013 27856047 10.1016/j.forsciint.2016.10.013

[14] Feldesman MR (1992) Femur/stature ratio and estimates of stature in children. Am J Phys Anthropol 87:447–459. 10.1002/ajpa.1330870406 1580352 10.1002/ajpa.1330870406

[15] Murray NJ, Spake L, Cervantes M et al (2024) New More Generic and Inclusive Regression Formulae for the Estimation of Stature from Long Bone Lengths in Children. Forensic Sci 4:62–75. 10.3390/forensicsci4010005

[16] Robbins Schug G, Gupta S, Cowgill LW et al (2013) Panel regression formulas for estimating stature and body mass from immature human skeletons: a statistical approach without reference to specific age estimates. J Archaeol Sci 40:3076–3086. 10.1016/j.jas.2013.02.025

[17] Ruff CB (2007) Body size prediction from juvenile skeletal remains. Am J Phys Anthropol 133:698–716. 10.1002/ajpa.20568 17295297 10.1002/ajpa.20568

[18] Smith SL (2007) Stature estimation of 3–10-year-old children from long bone lengths. J Forensic Sci 52:538–546. 10.1111/j.1556-4029.2007.00428.x 17456079 10.1111/j.1556-4029.2007.00428.x

[19] Hoppa RD, Gruspier KL (1996) Estimating diaphyseal length from fragmentary subadult skeletal remains: Implications for palaeodemographic reconstructions of a southern Ontario ossuary. Am J Phys Anthropol 100:341–354. 10.1002/(SICI)1096-8644(199607)100:3<341::AID-AJPA3>3.0.CO;2-X 8798992 10.1002/(SICI)1096-8644(199607)100:3<341::AID-AJPA3>3.0.CO;2-X

[20] Chu EY, Stull KE, Sylvester AD (2022) An alternative size variable for allometric investigations in subadults. Am J Biol Anthropol 179:471–480. 10.1002/ajpa.24617

[21] Ahmed AA (2013) Estimation of stature using lower limb measurements in Sudanese Arabs. J Forensic Leg Med 20:483–488. 10.1016/j.jflm.2013.03.019 23756519 10.1016/j.jflm.2013.03.019

[22] Menéndez Garmendia A, Sánchez-Mejorada G, Gómez-Valdés JA (2018) Stature estimation formulae for Mexican contemporary population: A sample based study of long bones. J Forensic Leg Med 54:87–90. 10.1016/j.jflm.2017.12.019 29331714 10.1016/j.jflm.2017.12.019

[23] Konigsberg LW, Frankenberg SR, Sgheiza V, Liversidge HM (2021) Prior Probabilities and the Age Threshold Problem: First and Second Molar Development. Human Biol 93:51. 10.13110/humanbiology.93.1.02 35338702 10.13110/humanbiology.93.1.02

[24] Sgheiza V (2022) Conditional independence assumption and appropriate number of stages in dental developmental age estimation. Forensic Sci Int 330:111135. 10.1016/j.forsciint.2021.111135 34883298 10.1016/j.forsciint.2021.111135

[25] Stull KE, L’Abbé EN, Ousley SD (2014) Using multivariate adaptive regression splines to estimate subadult age from diaphyseal dimensions. Am J Phys Anthropol 154:376–386. 10.1002/ajpa.22522 24782395 10.1002/ajpa.22522

[26] Stull KE, Chu EY, Corron LK, Price MH (2023) Mixed cumulative probit: a multivariate generalization of transition analysis that accommodates variation in the shape, spread and structure of data. R Soc Open Sci 10:220963. 10.1098/rsos.220963 36866077 10.1098/rsos.220963 PMC9974299

[27] Bogin B (2005) Patterns of human growth, 2. ed., reprint. Cambridge University Press, Cambridge

[28] Cameron N, Bogin B (2012) Human growth and development, 2nd edn. Elsevier/AP, London, UK

[29] Corron LK, Marchal F, Condemi S et al (2019) Integrating growth variability of the ilium, fifth lumbar vertebra, and clavicle with multivariate adaptive regression splines models for subadult age estimation. J Forensic Sci 64:34–51. 10.1111/1556-4029.13831 29852519 10.1111/1556-4029.13831

[30] Lottering N, Alston-Knox CL, MacGregor DM et al (2017) Apophyseal Ossification of the Iliac Crest in Forensic Age Estimation: Computed Tomography Standards for Modern Australian Subadults. J Forensic Sci 62:292–307. 10.1111/1556-4029.13285 27885641 10.1111/1556-4029.13285

[31] Ives R, Humphrey L (2017) Patterns of long bone growth in a mid-19th century documented sample of the urban poor from Bethnal Green, London, UK. Am J Phys Anthropol 163:173–186. 10.1002/ajpa.23198 28276055 10.1002/ajpa.23198

[32] Stull KE, Corron LK (2022) The Subadult Virtual Anthropology Database (SVAD): An accessible repository of contemporary subadult reference data. Forensic Sci 2:20–36. 10.3390/forensicsci2010003

[33] Hauspie R, Roelants M (2012) Adolescent growth. In: Cameron N, Bogin B (eds) Human Growth and Development, 2nd edn. Elsevier, pp 57–79

[34] Stull KE, Corron LK (2021) Subadult Virtual Anthropology Database (SVAD) Data Collection Protocol: Epiphyseal Fusion, Diaphyseal Dimensions, Dental Development Stages, Vertebral Neural Canal Dimensions. 10.5281/ZENODO.5348392

[35] Langley NR, Jantz LM, Ousley SD, Jantz RL (2016) Data collection procedures for forensic skeletal material 2.0. University of Tennessee and Lincoln Memorial University

[36] R Core Team (2024) R: A language and environment for statistical computing

[37] RStudio Team (2024) RStudio: Integrated development environment for R

[38] Rani Das K, RahmatullahImon AHM (2016) A brief review of tests for normality. AJTAS 5:5. 10.11648/j.ajtas.20160501.12

[39] Shapiro SS, Wilk MB (1965) An analysis of variance test for normality (complete samples). Biometrika 52:591–611. 10.1093/biomet/52.3-4.591

[40] Kendall MG (1949) Rank and Product-Moment Correlation. Biometrika 36:177. 10.2307/2332540 18132091

[41] Schaeffer MS, Levitt EE (1956) Concerning Kendall’s tau, a nonparametric correlation coefficient. Psychol Bull 53:338–346. 10.1037/h0045013 13336201 10.1037/h0045013

[42] Alexopoulos EC (2010) Introduction to multivariate regression analysis. Hippokratia 14:23–28 21487487 PMC3049417

[43] Massey FJ Jr (1951) The Kolmogorov-Smirnov test for goodness of fit. J Am Stat Assoc 46:68–78

[44] Colin Cameron A, Windmeijer FAG (1997) An R-squared measure of goodness of fit for some common nonlinear regression models. J Econom 77:329–342. 10.1016/S0304-4076(96)01818-0

[45] Giavarina D (2015) Understanding Bland Altman analysis. Biochem Med 25:141–151. 10.11613/BM.2015.015 10.11613/BM.2015.015 PMC4470095 26110027

[46] (1993) Daubert v. Merrel Dow Pharmaceuticals, Inc

[47] Grivas CR, Komar DA (2008) Kumho, Daubert, and the nature of scientific inquiry: Implications for forensic anthropology. J Forensic Sci 53:771–776. 10.1111/j.1556-4029.2008.00771.x 18489550 10.1111/j.1556-4029.2008.00771.x

[48] (1999) Kumho Tire Company, Ltd. v. Carmichael

[49] Lesciotto KM (2015) The impact of Daubert on the admissibility of forensic anthropology expert testimony. J Forensic Sci 60:549–555. 10.1111/1556-4029.12740 25716577 10.1111/1556-4029.12740

[50] Saunders S, Hoppa R, Southern R (1993) Diaphyseal growth in a nineteenth century skeletal sample of subadults from St Thomas’ church, Belleville, Ontario. Int J Osteoarchaeol 3:265–281. 10.1002/oa.1390030405

[51] Kemkes-Grottenthaler A (2005) The short die young: The interrelationship between stature and longevity—evidence from skeletal remains. Am J Phys Anthropol 128:340–347. 10.1002/ajpa.20146 15861421 10.1002/ajpa.20146

[52] Mummert A, Esche E, Robinson J, Armelagos GJ (2011) Stature and robusticity during the agricultural transition: Evidence from the bioarchaeological record. Econ Hum Biol 9:284–301. 10.1016/j.ehb.2011.03.004 21507735 10.1016/j.ehb.2011.03.004

[53] Ruff CB, Niskanen M (2018) Introduction to special issue: Body mass estimation — Methodological issues and fossil applications. J Hum Evol 115:1–7. 10.1016/j.jhevol.2017.09.011 29174414 10.1016/j.jhevol.2017.09.011

[54] Grabowski M, Hatala KG, Jungers WL, Richmond BG (2015) Body mass estimates of hominin fossils and the evolution of human body size. J Hum Evol 85:75–93. 10.1016/j.jhevol.2015.05.005 26094042 10.1016/j.jhevol.2015.05.005

[55] Wright LE, Vásquez MA (2003) Estimating the length of incomplete long bones: Forensic standards from Guatemala. Am J Phys Anthropol 120:233–251. 10.1002/ajpa.10119 12567377 10.1002/ajpa.10119

[56] Lee J-H, Kim YS, Lee U-Y et al (2014) Stature estimation from partial measurements and maximum length of lower limb bones in Koreans. Aust J Forensic Sci 46:330–338. 10.1080/00450618.2013.877078

[57] Fongkete I, Singsuwan P, Prasitwattanaseree S et al (2016) Estimation of stature using fragmentary femur and tibia lengths in a Thai population. Aust J Forensic Sci 48:287–296. 10.1080/00450618.2015.1052758

[58] Bidmos MA (2008) Estimation of stature using fragmentary femora in indigenous South Africans. Int J Legal Med 122:293–299. 10.1007/s00414-007-0206-2 17901969 10.1007/s00414-007-0206-2

[59] Cardoso HFV, Abrantes J, Humphrey LT (2014) Age estimation of immature human skeletal remains from the diaphyseal length of the long bones in the postnatal period. Int J Legal Med 128:809–824. 10.1007/s00414-013-0925-5 24126574 10.1007/s00414-013-0925-5

[60] Cameron N (1986) Standards for human growth - their construction and use. S Afr Med J 70:422–425 3490000

[61] de Onis M, Onyango A, Borghi E et al (2012) Worldwide implementation of the WHO Child Growth Standards. Public Health Nutr 15:1603–1610. 10.1017/S136898001200105X 22717390 10.1017/S136898001200105X

[62] Schillaci MA, Sachdev HPS, Bhargava SK (2012) Comparison of the maresh reference data with the who international standard for normal growth in healthy children. Am J Phys Anthropol 147:493–498. 10.1002/ajpa.22018 22282150 10.1002/ajpa.22018

[63] Langley NR (2016) The lateral clavicular epiphysis: fusion timing and age estimation. Int J Legal Med 130:511–517. 10.1007/s00414-015-1236-9 26253853 10.1007/s00414-015-1236-9

[64] López-Costas O, Rissech C, Trancho G, Turbón D (2012) Postnatal ontogenesis of the tibia. Implications for age and sex estimation. Forensic Sci Int 214:207.e1-207.e11. 10.1016/j.forsciint.2011.07.038 21862250 10.1016/j.forsciint.2011.07.038

[65] AAFS Standards Board (2019) Standard for stature estimation in forensic anthropology

[66] Heskes T (1996) Practical confidence and prediction intervals. Advances in Neural Information Processing Systems 9

[67] Ousley SD (1995) Should we estimate biological or forensic stature? J Forensic Sci 40:15381J. 10.1520/JFS15381J

[68] Wilson RJ, Herrmann NP, Jantz LM (2010) Evaluation of Stature Estimation from the Database for Forensic Anthropology. J Forensic Sci 55:684–689. 10.1111/j.1556-4029.2010.01343.x 20345794 10.1111/j.1556-4029.2010.01343.x

[69] Chou C-C, Iwasa Y, Nakazawa T (2016) Incorporating an ontogenetic perspective into evolutionary theory of sexual size dimorphism. Evolution 70:369–384. 10.1111/evo.12857 26768067 10.1111/evo.12857

[70] Gustafsson A, Lindenfors P (2009) Latitudinal patterns in human stature and sexual stature dimorphism. Ann Hum Biol 36:74–87. 10.1080/03014460802570576 19085512 10.1080/03014460802570576

[71] Ubelaker DH, DeGaglia CM (2017) Population variation in skeletal sexual dimorphism. Forensic Sci Int 278:407.e1-407.e7. 10.1016/j.forsciint.2017.06.012 28698063 10.1016/j.forsciint.2017.06.012

